# Nitrogen recovery from low-value biogenic feedstocks via steam gasification to methylotrophic yeast biomass

**DOI:** 10.3389/fbioe.2023.1179269

**Published:** 2023-05-30

**Authors:** Roghayeh Shirvani, Alexander Bartik, Gustavo A. S. Alves, Daniel Garcia de Otazo Hernandez, Stefan Müller, Karin Föttinger, Matthias G. Steiger

**Affiliations:** ^1^ Research Group Biochemistry, Institute of Chemical, Environmental and Bioscience Engineering, TU Wien, Vienna, Austria; ^2^ Doctoral College CO_2_Refinery, Faculty of Technical Chemistry, TU Wien, Vienna, Austria; ^3^ Research group Industrial Plant Engineering and Application of Digital Methods, Institute of Chemical, Environmental and Bioscience Engineering, TU Wien, Vienna, Austria; ^4^ Research Group Technical Catalysis, Institute of Materials Chemistry, TU Wien, Vienna, Austria

**Keywords:** methylotrophic yeast, ammonia, biodiesel scrubber, DFB gasification, biorefinery, sewage sludge, Komagataella phaffii (P. pastoris), Pichia pastoris

## Abstract

Carbon and nitrogen are crucial elements for life and must be efficiently regenerated in a circular economy. Biomass streams at the end of their useful life, such as sewage sludge, are difficult to recycle even though they contain organic carbon and nitrogen components. Gasification is an emerging technology to utilize such challenging waste streams and produce syngas that can be further processed into, e.g., Fischer-Tropsch fuels, methane, or methanol. Here, the objective is to investigate if nitrogen can be recovered from product gas cleaning in a dual fluidized bed (DFB) after gasification of softwood pellets to form yeast biomass. Yeast biomass is a protein-rich product, which can be used for food and feed applications. An aqueous solution containing ammonium at a concentration of 66 mM was obtained and by adding other nutrients it enables the growth of the methylotrophic yeast *Komagataella phaffii* to form 6.2 g.L^−1^ dry yeast biomass in 3 days. To further integrate the process, it is discussed how methanol can be obtained from syngas by chemical catalysis, which is used as a carbon source for the yeast culture. Furthermore, different gas compositions derived from the gasification of biogenic feedstocks including sewage sludge, bark, and chicken manure are evaluated for their ability to yield methanol and yeast biomass. The different feedstocks are compared based on their potential to yield methanol and ammonia, which are required for the generation of yeast biomass. It was found that the gasification of bark and chicken manure yields a balanced carbon and nitrogen source for the formation of yeast biomass. Overall, a novel integrated process concept based on renewable, biogenic feedstocks is proposed connecting gasification with methanol synthesis to enable the formation of protein-rich yeast biomass.

## 1 Introduction

The global nitrogen cycle is deeply affected by human activity, which impacts ecosystems and contributes to climate change (Steffen et al., 2015). Worldwide, the Haber-Bosch process accounts for approximately 2% of the world’s energy use to produce nitrogen fertilizer (Liu et al., 2020). Nitrogen in the reduced form of ammonia is a building block for essential biomolecules including amino acids and nucleic acids. In all biomass streams nitrogen can be found at different concentrations including low-value biomass streams like manure or sewage sludge. Nitrogen contained can partially be recovered when used as a plant fertilizer. Due to its hygienic risks and contamination with chemicals and heavy metals, legislation on landfilling of sewage sludge is tightening. Amongst other options thermal processes, like incineration, pyrolysis, or gasification can be used for sludge treatment, with incineration being the most common thermal process (Kacprzak et al., 2017). The incineration processes can be coupled with a preceding anaerobic digestion process to produce biogas (Amann et al., 2021; Zhao et al., 2023). During incineration nitrogen is mainly released as atmospheric N_2_ (about 94%), while also nitrogen oxides can be released in significant quantities. Especially, the primary greenhouse gas nitrous oxide (N_2_O) in quantities of up to 1.6% of total incoming nitrogen (Marias et al., 2015). In order to recover at least part of the nitrogen from sewage sludge, continuous thermal drying processes are being investigated (Deviatkin et al., 2019).

Another option for sludge treatment than incineration are thermal gasification processes (Schweitzer et al., 2018). Steam gasification is the process of thermally decomposing a solid fuel under high temperatures, with the addition of steam, to create a synthesis gas, or “syngas.” This syngas is composed mainly of hydrogen, carbon monoxide, carbon dioxide, and methane but the raw product gas is also loaded with tars, ammonia, water and other impurities. Dual fluidized bed (DFB) gasification is the most widely used form of steam gasification. This process uses two interconnected fluidized beds. A bubbling fluidized bed gasification reactor and a fast fluidized bed combustion reactor. In the gasification reactor, the endothermic gasification reactions of the solid fuel take place, while the combustion reactor provides the necessary heat to drive the gasification reactions.

The generated product gas can be used as syngas to produce valuable compounds like Fischer-Tropsch fuels (Gruber et al., 2021), synthetic natural gas (Thunman et al., 2018), methanol (Holmgren et al., 2012) or dimethyl ether (Landalv et al., 2014). However, gas cleaning is required to upgrade the raw product gas to a syngas suitable for synthesis. A biodiesel scrubber is typically used to separate tars, ammonia and water from the raw product gas. Since most of the fuel nitrogen is released as ammonia during steam gasification, an ammonium-rich water phase can be obtained from the biodiesel scrubber (Wilk, 2013; Bardolf, 2018). Nitrogen accumulating in this water phase is not recovered and needs to be removed during waste water treatment. Therefore, the scope of this work is to assess if an industrially used yeast strain is capable of utilizing this nitrogen source.

Methylotrophic yeasts like *Komagataella phaffii* (*Pichia pastoris)* are able to consume methanol as a sole carbon and energy source which makes them interesting microbial hosts for the sustainable production of biomass and proteins (Gasser et al., 2013; Peña et al., 2018) and nitrogen sources are a crucial factor during protein synthesis. Ammonia, ammonium salts, urea, and organic nitrogen, have already been used in different studies as nitrogen sources for yeast growth (Doran, 2012; Ye et al., 2017). In the late 1960s, Phillips Petroleum company developed processes to produce single-cell proteins (SCP) and developed the process around a strain of *P. pastoris*, which had high productivity (>10 g.L^−1^.h^−1^ dry cell weight) and cell densities (125–150 g.L^−1^ DCW) (Wegner, 1990). Methanol was considered as a source of carbon for methylotrophic yeasts at these early stages of the research. Although the protein product was feed-grade, it was economically unfavorable to manufacture protein from fossil methanol when compared with protein derived from soybeans and other sources (Johnson, 2012; Ritala et al., 2017). Today SCP production has different markets and applications (Ritala et al., 2017) and yeast derived SCP is used as a protein rich feed ingredient for instance in aquafeeds (Agboola et al., 2021). The production of SCP from methanol is also returning to the spotlight, since this carbon source can be produced without relying on agricultural inputs. A few studies have focused on the development of methods for upgrading syngas to products in a microbial fermentation process using the direct conversion of CO, CO_2_, H_2_, or methane to bioproducts in anaerobic fermentation of acetogenic or methanotrophic bacteria, while to our knowledge, no studies have focused on the conversion of syngas to methanol followed by aerobic fermentation of methylotrophic yeasts to bioproducts like SCP (Frazão and Walther, 2020; García Martínez et al., 2022).

Besides its role as a growing medium for methylotrophic yeasts, methanol is one of the most relevant building block compounds in the chemical industry. Despite being primarily produced from fossil resources, green methanol derived from CO_2_ and hydrogen is on the rise (Sollai et al., 2023). In a methanol economy, this C_1_ compound plays an important role as a raw material, energy storage molecule, and transportation fuel (Olah et al., 2008; Olah, 2013). Over the last decades, it has been industrially produced from the catalytic conversion of syngas, which consists of a mixture of H_2_, CO and CO_2_. The process is typically conducted with a Cu/ZnO/Al_2_O_3_ catalyst at 200–300°C and 50–100 bar (Behrens et al., 2012). Under these conditions, the following reactions must be considered:

$$\begin{array}{cc} CO+2H_{2}\to {CH}_{3}OH & -90.7\;kJ.{mol}^{-1} \end{array}$$ (R1)

$$\begin{array}{cc} {CO}_{2}+3H_{2}\to {CH}_{3}OH+H_{2}O & -49.5\;kJ.{mol}^{-1} \end{array}$$ (R2)

$$\begin{array}{cc} CO+H_{2}O\;\to \;{CO}_{2}+2H_{2} & -41.2\;kJ.{mol}^{-1} \end{array}$$ (R3)

Although the overall reaction can be described as CO hydrogenation (R1), it has been shown that the combination of CO_2_ hydrogenation (R2) with the water-gas shift reaction (R3) is actually the kinetically favored pathway in the most commonly used catalysts (Studt et al., 2015). Industrial methanol production has been thoroughly optimized to operate from fossil fuel feedstocks, in order to adjust the syngas composition to an optimal molar ratio of (H_2_-CO_2_)/(CO + CO_2_) around 2. However, biomass-derived syngas often presents distinct properties, such as a higher CO_2_ content. Considering the adverse effects of CO_2_ and other impurities in syngas, environmentally friendly and efficient methanol production by steam gasification of biomass would present additional challenges, which are addressed here.

Here we present a new strategy for an integrated process coupling steam gasification with the production of yeast biomass by a yeast culture. By using a biodiesel scrubber, an ammonium-containing water phase is generated downstream gasification. The growth of yeast in the ammonium-containing water phase is demonstrated using methanol as a carbon source. Methanol can be produced from syngas mixtures using adaptations to methanol catalysis, which are discussed. Finally, different biogenic feedstocks are compared showing the potential to produce a valuable yeast biomass stream while retaining a maximal amount of nitrogen.

## 2 Material and methods

### 2.1 Gasification procedure

Steam gasification of softwood pellets was carried out in a 100 kW_thermal_ advanced DFB gasifier with an 80/20 wt.-% mixture of olivine/limestone as bed material. Around 20 kg/h of softwood pellets were converted to a product gas at 840°C in a steady-state operation for 8.5 h. Relevant parameters of the softwood pellets for gasification are provided in Supplementary Table S1. After cooling, a slipstream of the product gas was directed to a particle filter and a biodiesel scrubber, while the rest was combusted in a post-combustion chamber. The biodiesel scrubber was operated at 18°C as a randomly-packed absorption column with rapeseed methyl ester (RME or biodiesel) as solvent. A phase separator connected to the biodiesel scrubber continuously separated the loaded RME into an oily RME phase, an emulsion phase, and a water phase. The oily RME phase was continuously recirculated to the scrubber, while the emulsion and water phases were collected in the phase separator for further analysis. The operating parameters of the DFB gasifier and the biodiesel scrubber are documented in the Supplementary Table S2. A detailed description of the gasifier in general can be found in literature (Schmid et al., 2021).

To close the nitrogen balance over the gasifier and the scrubber, the nitrogen content of the fuel and the ammonia concentration in the raw product gas and the cleaned product gas downstream the scrubber were measured. Additionally, the water content and the tar content of the raw product gas were determined. Sampling was performed following the tar protocol (DIN CEN/TS 15439) and is documented in (Wolfesberger-Schwabl, 2013). The main gas components like H_2_, CO, CO_2_, and CH_4_ were continuously measured with Rosemount NGA-2000 modules.

### 2.2 Treatment of the sample obtained from the biodiesel scrubber

From the biodiesel scrubber, a sample was taken; the oily phase was discarded using a separatory funnel and the aqueous phase was separated in a few steps. First, the aqueous phase was filtered through filter paper followed by centrifugation at 10,000 g for 10 min. In the last step the sample was steril filtered through a 0.2 µm steritop filter (Merckmillipore, Burlington, United States). The ammonium containing water solution was used as a nitrogen source to prepare a cultivation media.

### 2.3 Strain and media

*Komagataella phaffii (P. pastoris) CBS7435* was used as a yeast strain in this study. The reference cultivation media; M2 Citrate buffered media, contained 3.15 g (NH_4_)_2_HPO_4_, 0.49 g MgSO_4_.7H_2_O, 0.80 g KCl, 0.0268 g CaCl_2_.2H_2_O, 22.0 g citric acid monohydrate, 4 mL biotin (0.1 g.L^−1^), and 1.47 mL trace salt solution (PTM0) per liter.

Trace salt solution (PTM0) contained; 5 mL sulphuric acid (95%–98%), 65 g FeSO_4_.7H_2_O, 20 g ZnCl_2_, 6 g CuSO_4_.5H_2_O, 3.36 g MnSO_4_.H_2_O, 0.82 g CoCL_2_.6H2O, 0.2 g Na_2_MoO_4_.2H_2_O, 0.08 g NaI, 0.02 g H_3_BO_3_ per liter.

The final pH of the media was adjusted to pH 6 by adding potassium hydroxide pellets. The purified biodiesel scrubber solution was used to prepare the GNM2 medium (**G**asification **N**itrogen **M**2 media), and ammonium recovered via DFB steam gasification was substituted for (NH_4_)_2_HPO_4_ as the nitrogen source. To compensate for phosphate, 3.2 g of KH_2_PO_4_ was added. Table 1 summarizes the main differences between various cultivation media.

**TABLE 1:** Media compositions used in this study.

Media	M2	GNM2	GNM2-MeOH	GN-M2+MeOH
Carbon source	Methanol	Methanol	-	Methanol
Nitrogen source	(NH_4_)_2_HPO_4_	NH_4_^+^ in water phase from gasification		
Other media components	-	KH_2_PO_4_		-
	MgSO_4_, KCl, CaCl_2_, citric acid, sulphuric acid, FeSO_4_, ZnCl_2_, CuSO_4_, MnSO_4_, CoCL_2,_ Na_2_MoO_4_, NaI, H_3_BO_3_, Biotin			
Solvent	Water	Water phase from gasification		

### 2.4 Biocultivation conditions

A single yeast colony from a YPD agar plate (1% yeast extract, 2% peptone, and 2% glucose, 1.5% bacteriological agar) was inoculated into a 250 mL shake flask containing 50 mL of YPD (1% yeast extract, 2% peptone, and 2% glucose) as a preculture. The cells were grown overnight at 28°C and 200 rpm in an incubator shaker (Infros HT, Bottmingen-Basel). Main cultivation media were inoculated with the overnight-grown cultures to each 500 ml shake flask, covered with cotton wools, containing 50 mL of cultivation media. The cultures were grown at 28°C and 200 rpm. The cultivations were fed with 5–10 g.L^−1^ methanol at different time points.

### 2.5 Biomass measurements

During cultivation, a spectrophotometer was used to monitor optical density at 600 nm (OD_600_) (Spectra max, plus 384, Downingtown, PA). All the measurements were done in technical triplicates. In order to measure the dry cell weight (DCW), 45 mL of cultivation supernatant was centrifuged at 5,000 *g* for 5 min. The cell pellet was washed twice with distilled water followed by centrifugation and transferred to a pre-weighed 50 ml falcon tube. After freezing at −80°C the cell pellet was placed in a freeze dryer (FreeZone 2.5, Labconco, MO) for 48–72 h. The weight of the obtained dry biomass was determined on a fine balance. Dry cell weight values were correlated with OD_600_ measurements. A dry cell weight (DCW) of 0.389 g.L^-1^ yields an OD_600_ of 1. The process yields were calculated covering the entire cultivation.

### 2.6 Nitrogen, ammonium, cyanate, and methanol measurements

Samples in 2 mL tubes were collected during the cultivation at defined time points. The supernatant was separated by centrifugation at 16,000 *g* for 10 min. The culture supernatants were used for nitrogen, ammonium and methanol measurements.

The ammonium concentration in different samples, including treated samples from the biodiesel scrubber and culture supernatants, were analyzed using an ammonium measurement kit (Roche Diagnostics GmbH, Germany) in a Cedex Bio Analyzer (I&L Biosystems GmbH, Germany).

Methanol in the culture supernatant was quantified by HPLC analysis on an LC-20 AC system equipped with an RID detector operated at 50°C (Shimadzu, Kyoto, Japan) and quantified using standards of analytical grade. HPLC separation on an Aminex HPX-87H, 300 × 7.8 mm column (Bio-Rad, Hercules, California, USA) was carried out using a 5 mM H_2_SO_4_ mobile phase at a flow rate of 0.6 ml min^-1^ at 60°C.

Total nitrogen measurements were done using a Eurovector EA 3000 CHNS-O Elemental Analyzer and cyanate was characterized by capillary (free zone) electrophoresis by the Microanalytical Laboratory, University of Vienna, Austria.

### 2.7 Calculation of methanol synthesis from syngas mixtures

Considering steam gasification with different biomass fuels presented in this work, the maximum methanol yield from a conventional methanol synthesis approach is estimated in each case. The estimation assumes carbon capture of excess CO_2_ and other unwanted species from the product gas and subsequent conversion of H_2_, CO and CO_2_ in a 2.3: 1: 0.1 ratio, which refers to a stoichiometric (H_2_-CO_2_)/(CO + CO_2_) ratio of 2 and a low CO_2_:CO ratio of 0.1 (Sahibzada et al., 1998).

## 3 Results

### 3.1 Dual fluidized bed gasification converts a biogenic feedstock into a product gas and an ammonium containing water phase

**FIGURE 1:**
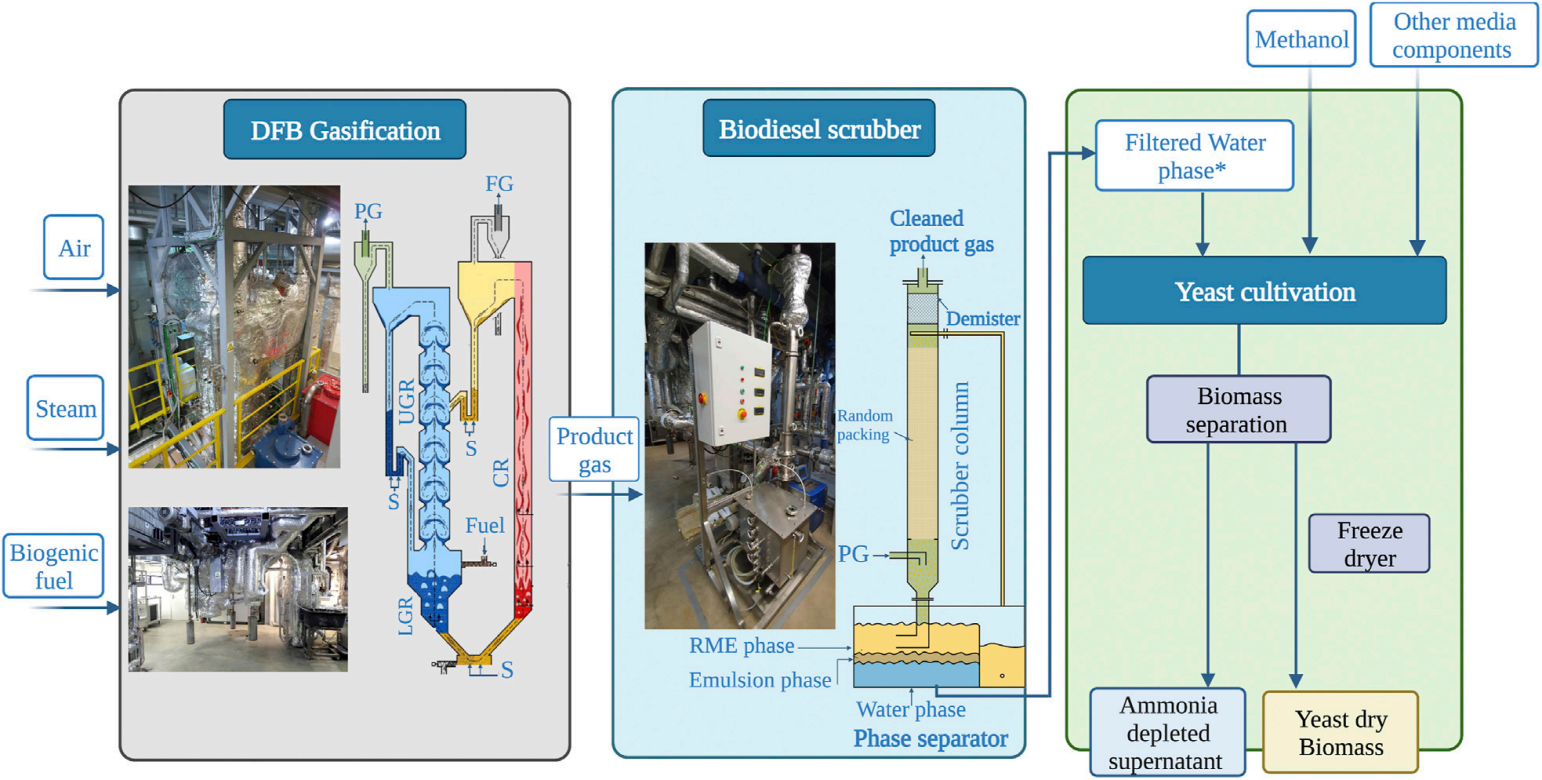
The integrative workflow employed in this study; shows how DFB steam gasification (left panel) is connected with a biodiesel scrubber (middle panel) yielding an aqueous phase, which can be used to prepare a medium for yeast cultivation (right panel). S; steam, PG; product gas, FG; flue gas, CR; combustion reactor, LGR; lower gasification reactor, UGR; upper gasification reactor. * Water fraction containing 65.69 mmol.L^−1^ ammonium.

A DFB gasifier was used to produce a product gas by steam gasification from solid, biogenic fuels. The primary product gas was treated in a biodiesel scrubber to remove tars, water and water-soluble impurities from the gas stream. Ammonium is a water-soluble compound that can be dissolved in the separated water phase. The setup is depicted in Figure 1. The cleaned product gas exiting the biodiesel scrubber has a syngas composition as reported in Table 3. The biogenic fuel used were softwood pellets, which had a nitrogen content of 0.2 wt.-%_daf_, which resulted in an ammonia concentration in the product gas of 570 ppm_v,db_. Considering the amount of water captured in the biodiesel scrubber, a calculated ammonium concentration of 63.4 mmol.L^−1^ is expected in the water-phase based on a measured ammonia separation efficiency of 95% from the product gas. This value corresponded well with the ammonium concentration of 65.7 mmol.L^−1^ which was directly measured in the aqueous phase of the treated samples from the biodiesel scrubber. This ammonium concentration is in the range of media used for yeast cultivation like the synthetic M2 media with 48 mmol.L^−1^ (Delic et al., 2013). Due to this nitrogen concentration, we explored the feasibility of using this aqueous phase of the biodiesel scrubber as a methylotrophic yeast growth medium.

### 3.2 Recovered ammonium from gasification can be utilized by yeast as a nitrogen source

**FIGURE 2:**
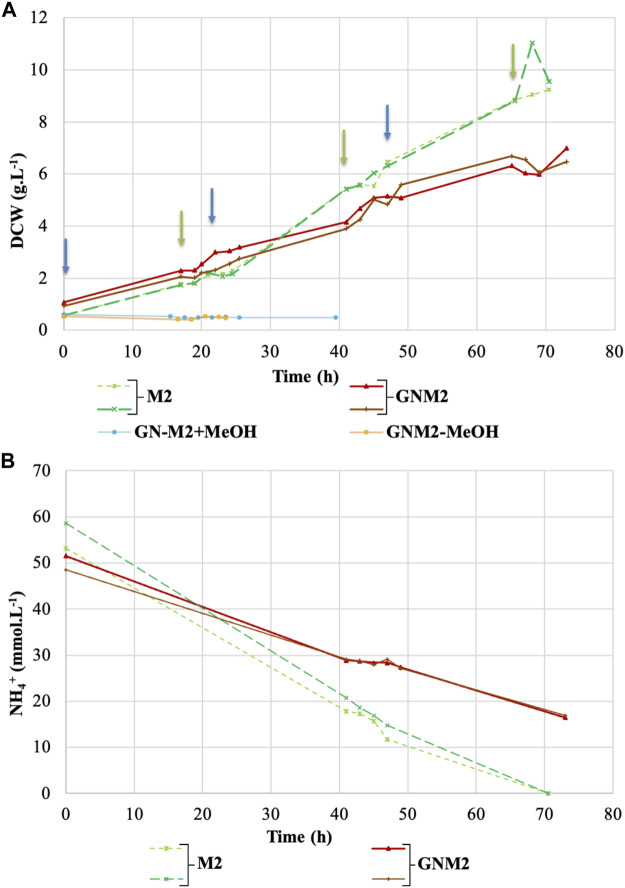
Growth profile and cultivation data of the *K. phaffii* in media containing nitrogen and water from a DFB gasification. **(A)** The DCW represents the yeast growth in M2 Media (green dashed lines), GNM2 media (red solid lines), GN media without M2 salts (blue line), and GN media without methanol (orange line). **(B)** Ammonium consumption during the cultivation time in M2 Media (green dashed lines) and GNM2 media (red lines) was shown. The arrows show methanol feeding time points: 5 g.L^−1^ (blue arrow) and 10 g.L^−1^ (green methanol). Results from duplicate experiments are shown in the same color.

A methylotrophic yeast was tested on different media compositions created from the water phase of the biodiesel scrubber. There was an addition of essential nutrients as well as methanol as a carbon source, but no additional ammonium source was added and also the water was directly coming from the biodiesel scrubber (GNM2 media—**G**asification **N**itrogen **M**2 media). As a control condition a standard synthetic M2 media was used. Two versions of the GNM2 media were tested. One without an additional carbon source (GNM2-MeOH) and one without additional nutrient salts (GN-M2+MeOH). An overview of the media compositions is provided in Table 1. All media were inoculated with *K. phaffii* and the results from the growth tests are shown in Figure 2A. No growth was observed for the GN-M2+MeOH media and the GNM2-MeOH. Growth was measured for the GNM2 media and the positive control M2 media. The biomass reached 6.2 g.L^−1^ in GNM2 media after 73 h and 8.3 g.L^−1^ in M2 media after 70.5 h. For the latter two media the ammonium concentration was followed over time, which is presented in Figure 2B. The entire ammonium was utilized after 70.5 h in case of the M2 media, whereas 16.7 mmol.L^−1^ were present in the GNM2 media at the end of the cultivation. Table 2 displays a summary of the data from the cultivations. Yield parameters on the carbon and nitrogen source were calculated for the entire cultivation period. The results showed that the obtained biomass yields on methanol are comparable between the two media M2 and GNM2. The biomass yield on ammonium appeared to be higher on the GNM2 medium compared to the M2 medium.

**TABLE 2:** Biomass formation and nitrogen consumption on M2 and GNM2 media with *K. phaffii*.

	M2	GNM2
Biomass formed (g_DCW_.L^-1^)	8.32 ± 0.60	6.18 ± 0.46
Total ammonium uptake (g_NH_4_^+^_.L^-1^)	1.00 ± 0.04	0.60 ± 0.03
Biomass yield on methanol [g_DCW_.g_MeOH^-1^_]	0.20 ± 0.01	0.21 ± 0.00
Biomass yield on ammonium [g_DCW_.g_[NH_4_^+^] ^−1^_]	8.22 ± 0.020	(10.33 ± 1.3)^app^

Ammonium was the only nitrogen source present in the chemically defined M2 medium. In the GNM2 sample a total nitrogen analysis revealed that ammonium accounts for 82% of the total nitrogen (0.86 ± 0.06 g.L^−1^ N) presented in the GNM2 sample. Additional analysis of the initial water phase from the biodiesel scrubber showed that it also included 0.15 mg.L^−1^ cyanate. In order to investigate the effect of cyanate on the growth of *K. phaffii,* the yeast was grown on the minimal media agar supplemented with sodium cyanate (NaOCN). *K. phaffii* can grow in the presence of sodium cyanate in the range of 2.5–10 mM as a sole nitrogen source (Supplementary Figure S1).

### 3.3 Methanol can be obtained from the product gas to produce yeast biomass

Methylotrophic yeast can grow on the C1 carbon source methanol, which can be obtained by chemical catalysis from syngas mixtures. Here it was shown, that a methylotrophic yeast can be grown directly in an aqueous phase obtained from the biodiesel scrubber, which provides both nitrogen and water for the biotechnological process. The next step was to determine which fraction of carbon can be directly produced from the biogenic feedstock. Data from biogenic feedstocks having different nitrogen contents were available for the DFB gasification process. In Table 3, the data of the nitrogen content and the respective product gas composition is provided. Based on the ammonia and water content in the product gas the theoretical ammonium concentration in the biodiesel scrubber can be determined together with the amount of water recovered there. It can be seen that the lowest ammonium loading is obtained from softwood pellets, which was used in this study to prepare the GNM2 media. The highest ammonium concentration of almost 9 M is calculated for chicken manure. Next the maximal biomass yield on the ammonium source was calculated assuming no limitation in the carbon source. With the biogenic feedstock composition also the product gas composition is changing. Based on the gas composition the methanol synthesis yield was calculated, which was used to determine the maximal biomass yield on the carbon source assuming no limitation in the nitrogen source. The biomass yield values are presented in Table 3 and for each feedstock it is determined if the carbon or the nitrogen source is limiting. Nitrogen is limiting for softwood pellets, while carbon is limiting for chicken manure rich in nitrogen. Interestingly, for the combination of bark and chicken manure an almost balanced composition of carbon and nitrogen source is produced showing that up to 21% of the feedstock can be converted into yeast biomass. Assuming that all ammonium is converted into biomass it was calculated how much methanol is generated or required for the process. This shows that up to 640 kg yeast biomass can be produced per ton of chicken manure requiring an additional 2.1 tons of methanol.

**TABLE 3:** Data of the DFB gasification of different biogenic feedstocks yielding methanol and biomass in an integrated biorefinery concept. daf.dry and ash free, db.dry basis, v.volumetric.

Fuel	Softwood pellets **(this study)**	Bark (Schmid et al. (2021))	Rice husk (Schmid et al. (2021))	Bark + chicken manure (70/30 wt%) (Schmid et al. (2021))	Sewage sludge (Schmid et al. (2019))	Chicken manure (Schmid et al. (2021))
N in fuel [wt.-%_daf_]	0.2	0.342	0.554	1.964	3.46	5.509
NH_3_ in product gas [ppm_v,db_]	570	3,300	7,600	23,800	46,000	73,200
Product gas yield [Nm^3^_db_.kg_fuel,db_^-1^]	1.42	1.34	1.01	1.25	0.70	1.13
Theoretical NH_4_^+^ concentration [mmol.L^-1^]	63.4	312.4	550.6	2,917.5	1,504.2	8,941.9
Theoretical water amount [kg_water_.kg_fuel,db_^-1^]	0.528	0.603	0.593	0.456	0.928	0.454
Product gas composition [vol.-%_db_]						
H_2_	44.6	51.9	43.1	43.8	32.5	40.1
CO	21.8	14.7	18	23.5	12.6	21
CO_2_	20.9	22.4	23.6	19.9	33.5	19.8
CH_4_	9.2	8.1	11	8	10.8	8.4
N_2_	2.5	1.6	1	1.2	2.2	1.3
C_2_H_4_	0.9	0.83	2.5	1.07	2.3	2.05
Product gas to MeOH conversion [vol%]	66	50	61	65	43	60
Max Methanol yield (available product gas) [kg_methanol._kg_fuel,db_^-1^]	1.14	0.81	0.75	1.00	0.36	0.82
Max Biomass yield (available methanol) [kg_biomass_ db.kg_fuel,db^-1^_]	0.25	0.18	0.17	0.22	0.08	0.18
Max Biomass yield (available NH_4_^+^) [kg_biomass_ db.kg_fuel db^-1^_]	0.01	0.03	0.05	0.21	0.22	0.64
Methanol Excess [kg.kg_fuel db^-1^_]	1.12	0.68	0.52	0.05	−0.64	−2.08
Limiting nutrient (N - nitrogen source, C- carbon source)	N	N	N	-	C	C

## 4 Discussion

### 4.1 Nitrogen recovery to yeast biomass

The main objective of gasification is to produce valuable syngas, which can be converted into a range of different chemicals. However, biogenic feedstocks always carry a certain fraction of nitrogen, which needs to be separated from the gas stream and accumulates in the water phase of the biodiesel scrubber as ammonium. The DFB gasification of softwood pellets yielded ammonia in the gas phase which was measured and used to determine the nitrogen loading in the water phase of the biodiesel scrubber. Interestingly this value deviated only by 3% from the actual measured ammonium concentration, which indicates that the installed scrubber is highly efficient in cleaning ammonia from the gas stream.

After phase separation, the slightly turbid water fraction obtained from the scrubber can be clarified by centrifugation and filtration in order to obtain an optically transparent, light amber solution. This clarification was carried out to avoid disturbing the measurements of the growth by optical density measurements and to accurately determine the cell dry weight at the end of the cultivation. Precipitates may be caused by tars solubilized in the biodiesel fraction; it can be tested in the future to see how yeast cells respond to it.

In the cleared aqueous phase supplemented with methanol and other media components (GNM2 media) it was possible to grow *K. phaffii* to cell densities, which were only 25% below the values obtained on a chemically defined M2 medium. The control condition containing methanol, but no additional media components (GN-M2+MeOH), showed no growth, indicating that other essential nutrients are missing in the biodiesel scrubber fraction. This is not surprising as non-volatile biomass components like phosphate and metal ions are accumulating in the ash and are not carried over to the scrubber (Parés Viader et al., 2017). Also, in the other control condition containing all media components except methanol (GNM2-MeOH) no growth was observed, which demonstrates that no readily accessible carbon source for the growth of *K. phaffii* is present. This data is in accordance with the comparable biomass yield data on methanol comparing M2 with GNM2 media. The biomass yield calculated on the known nitrogen source ammonium appeared higher on the GNM2 media compared to M2 medium. This indicated the presence of an additional nitrogen source. Further analysis showed that up to 18% of the total nitrogen is not ammonia but another nitrogen species. HCN is a likely candidate for a nitrogen source as it was detected in the DFB product gas at levels of about 10% of the ammonia in the product gas (Wilk, 2013). However, to this end a direct measurement of cyanide was not possible due to the sample matrix effects of the biodiesel scrubber phase, but low levels of cyanate were detected. The effect of cyanide on *K. phaffii* is not described but a certain toxicity on the respiratory chain can be assumed. Cyanide can be oxidized abiotically to cyanate which can be used as a nitrogen source by yeast including *Komagataella pastoris* a closely related strain to the one used in this study (Linder, 2019).We show that *K. phaffii* is also able to grow on cyanate as a sole nitrogen source. This makes it likely that cyanate is used beside ammonium as a nitrogen source during growth on the GNM2 medium.

Cyanide but also other compounds like tars might contribute to the reduced growth rate of the *K. phaffii* strain in GNM2 media compared to M2 media. A laboratory evolution approach containing inhibitory substances, however, can address this reduced growth rate (Dragosits and Mattanovich, 2013). Nevertheless, the influence of tars can be tested using feedstocks, which produce a higher tar content than softwood pellets. Feedstocks with a higher nitrogen content yield a higher ammonium concentration which likely leads to saponification of the biodiesel ester (RME), reducing the amount of ammonium available in the water phase. Adding a tenside could prevent this problem as was shown before (Bardolf, 2018).

Under culture conditions in a chemostat, the biomass yield on methanol of the yeast *K. phaffii* can reach up to 0.40 (g_Biomass_.g_MeOH_^-1^) (Tomàs-Gamisans et al., 2018), which is higher than the yield obtained in this study. This is mainly due to the controlled condition in a bioreactor cultivation such as DO (dissolved oxygen), pH and continuous carbon and nitrogen feeding. Besides, cultivations in lab shaking flasks are more likely to result in methanol evaporation. The average protein content of *K. phaffii* cells grown on methanol was found to be 50%, which is considerably higher than the average protein content of cells grown on glycerol (41%), and glucose (37%) (Tomàs-Gamisans et al., 2018). This is promising for SCP production where a high protein content of the product is important.

### 4.2 Development of a concept for an integrated process combining gasification, methanol synthesis and yeast cultivation

**FIGURE 3:**
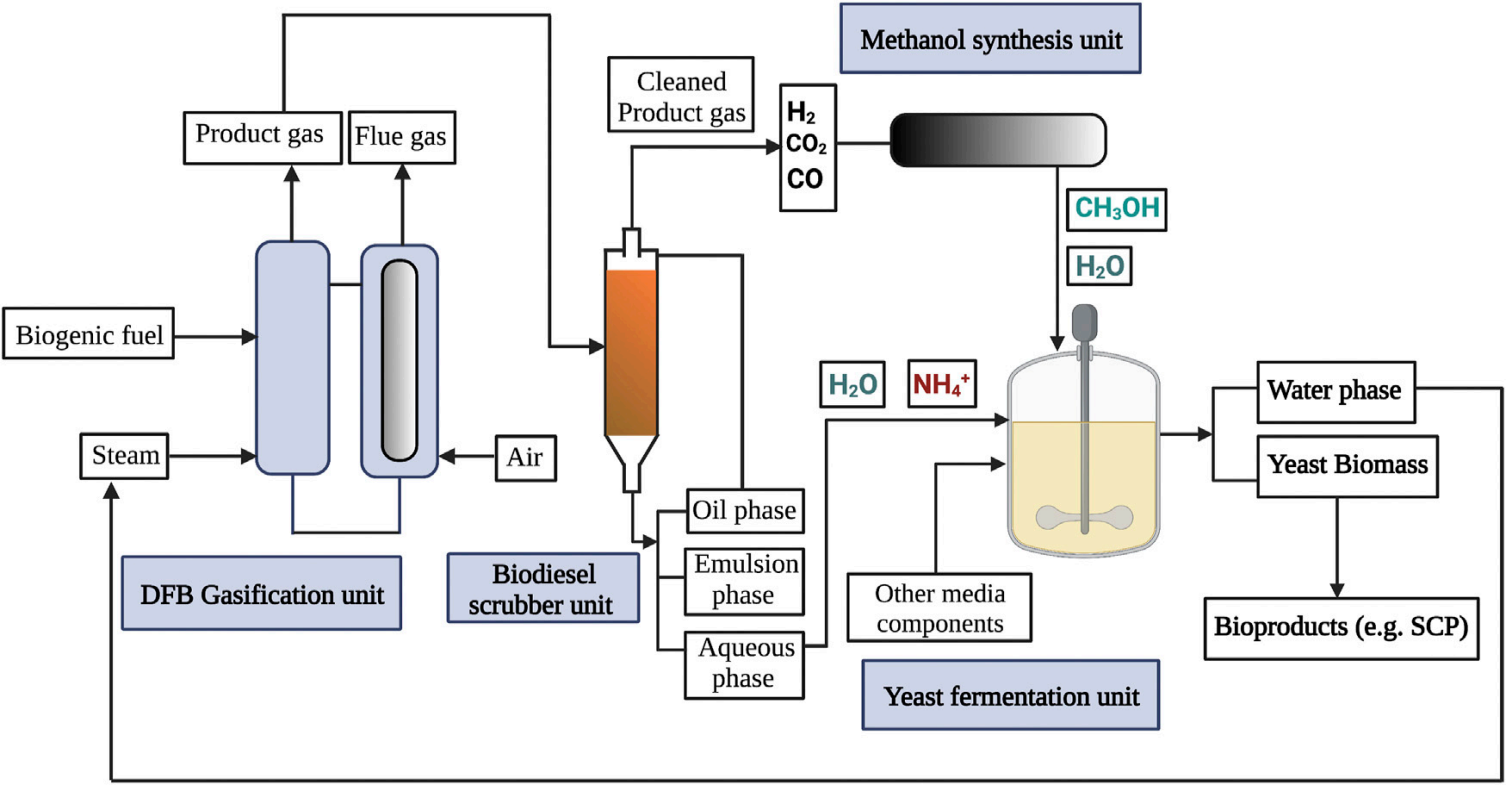
From biogenic fuels to yeast biomass, a proposed integrated approach of gasification; gas cleaning, methanol synthesis and cultivation is presented. DFB gasification is a versatile unit operation to convert biogenic feedstocks into a product gas. The ammonium-containing aqueous phase of the biodiesel scrubber unit is used to cultivate methylotrophic yeast. The carbon source methanol is produced from the syngas obtained from the gasification. After cell separation the ammonium depleted water phase can be reused for steam generation closing the water cycle.

Considering that nitrogen recovered from steam gasification of biomass can be effectively utilized in the growing medium of methylotrophic yeast for the production of SCP, the integration of these processes could be further accomplished in a biorefinery approach. In this concept, product gas from biomass gasification can be further upgraded into methanol, which would be in turn introduced as a carbon source for yeast fermentation, as illustrated in Figure 3.

One of the most critical challenges in integrating biomass gasification with methanol production is the high CO_2_ content in the product gas. Although the CO_2_ hydrogenation reaction is directly involved in methanol synthesis, abundant CO_2_ can lead to an excessive prominence of CO_2_ hydrogenation and the reverse water-gas shift reaction, thereby producing large amounts of H_2_O together with methanol. In this scenario, in addition to the less favorable reaction thermodynamics, the high-water content poses a major constraint to catalyst stability, as it promotes sintering and segregation of Cu and ZnO particles (Fichtl et al., 2015), thus deactivating the catalyst during continuous operation (Wu et al., 2001). Therefore, integrating biomass gasification with a methanol synthesis unit would necessarily involve gas conditioning steps. This is because excess CO_2_ is captured from product gas and subsequently stored or utilized in other processes. In addition, the separation of hydrocarbons, tar and other common trace gases such as H_2_S should also be considered (Brown, 2019).

In the presence of syngas with an optimal CO: CO_2_: H_2_ content after gas conditioning, industrial methanol synthesis typically features selectivity above 99%, although single-pass conversion is limited to 20% due to thermodynamics and catalyst stability considerations. In order to maximize the conversion rate, unreacted syngas is often recompressed and recycled into the reactor (Frolov et al., 2023).

The most readily available techniques to capture excess CO_2_ from syngas for methanol synthesis involve physical (Yamada, 2021) or chemical absorption methods (Giuliano et al., 2020). Further methods such as Pressure Swing Adsorption could simultaneously capture CO_2_ and CH_4_ from the product gas, in order to achieve the optimal ratio between H_2_, CO, and CO_2_ (Puig-Gamero et al., 2018).

As a promising alternative to carbon capture and storage, direct utilization of the CO_2_-rich stream within the methanol production process could greatly improve carbon efficiency and methanol yield. For example, it has been suggested that the negative effect of water due to high CO_2_ content may be mitigated by introducing intermediate condensation steps (Lacerda de Oliveira Campos et al., 2022), membrane reactors (Gallucci et al., 2004) and more suitable catalysts for CO_2_-rich conditions (Asthana et al., 2022). In another recently proposed concept, the reverse water-gas shift reaction was combined with water sorption and methanol synthesis in the same reactor, converting excess CO_2_ into CO in order to enhance methanol production (Peinado et al., 2021). Furthermore, direct CO_2_ hydrogenation to methanol may become a key technique to this integrated process with the recent progress on selective and stable catalysts, such as ZnO-ZrO2 (Wang et al., 2017), In_2_O_3_ (Wang et al., 2021) and MoS2 (Hu et al., 2021). In the near future, these emerging CO_2_ utilization concepts and technologies may offer viable strategies to minimize the carbon footprint of such integrated biorefinery processes. Recovering both nitrogen and carbon from a biogenic feedstock makes this integrated process an attractive alternative compared to processes where ammonia is recovered from a biogas slurry using *Candida utilis*. In this approach comparable biomass titers are achieved, but glucose had to be provided as an external carbon source (Ding et al., 2023).

## 5 Conclusion

In this study, we utilized an ammonium-containing side stream of a DFB gasification to produce a growth medium for the methylotrophic yeast *K. phaffii*. As a carbon source methanol was used which can be obtained from syngas via chemical catalysis. An aqueous solution containing ammonium at a concentration of 66 mM was obtained from the biodiesel scrubber, which was used as source of nitrogen in a cultivation media by adding other nutrients. It was shown that yeast can reach a dry cell weight of 6.2 g.L^-1^ on this media by utilizing the nitrogen provided by the gasification stream. Based on the experimental biomass yield data, different biogenic feedstocks were compared for their potential to produce yeast biomass. For this purpose, the product gas composition was determined after gasification and was used to calculate its conversion to methanol. As a result of this comparison, softwood pellets evaluated in this study make a carbon surplus while a mixture of bark and chicken manure would provide yeast with a balanced nitrogen and carbon source. Yeast biomass is a protein-rich product, which can be utilized as a single cell protein for feed applications. The integrative approach presented offers a sustainable process with multiple applications. It enables a technically feasible solution to partially recover carbon and nitrogen in a reduced form from low-value biogenic feedstocks like sewage sludge. This is in stark contrast to the incineration process that releases both nitrogen and carbon into the atmosphere. In addition, process water from gasification can be directly used to prepare a sterile (germ-free) growth medium for biotechnological applications, which saves water and energy when directly coupled. The process does not require agricultural resources when using sewage sludge as a fuel and can provide an alternative source of protein for feed production.

## Data Availability

The original contributions presented in the study are included in the article/Supplementary Material, further inquiries can be directed to the corresponding author.
