# Supplementary material for: Nitrogen recovery from low-value biogenic feedstocks via steam gasification to methylotrophic yeast biomass
Source: Front Bioeng Biotechnol. 2023 May 30;11:1179269. doi: 10.3389/fbioe.2023.1179269 (PMC10289294; doi:10.3389/fbioe.2023.1179269)
Supplement: Supplementary file 1 [file DataSheet1.PDF]

Supplementary Material

Nitrogen recovery from low-value biogenic feedstocks via steam gasification to yeast biomass

Roghayeh Shirvani[1,2], Alexander Bartik[2,3], Gustavo A.S. Alves[2,4], Daniel Garcia de Otazo Hernandez [1], Stefan Müller[2,3], Karin Föttinger [2,4], Matthias G. Steiger[1,2*]

[1]Research group Biochemistry, Institute of Chemical, Environmental and Bioscience Engineering, TU Wien, Vienna, Austria

[2]Doctoral College CO_2Refinery, Faculty of Technical Chemistry, TU Wien, Vienna, Austria

[3]Research group Industrial Plant Engineering and Application of Digital Methods, Institute of Chemical, Environmental and Bioscience Engineering, TU Wien, Vienna, Austria

[4]Research group Technical Catalysis, Institute of Materials Chemistry, TU Wien, Vienna, Austria

*** Correspondence: Dr. Matthias Steiger** matthias.steiger@tuwien.ac.at

1 Supplementary Data

1.1 Supplementary Figure

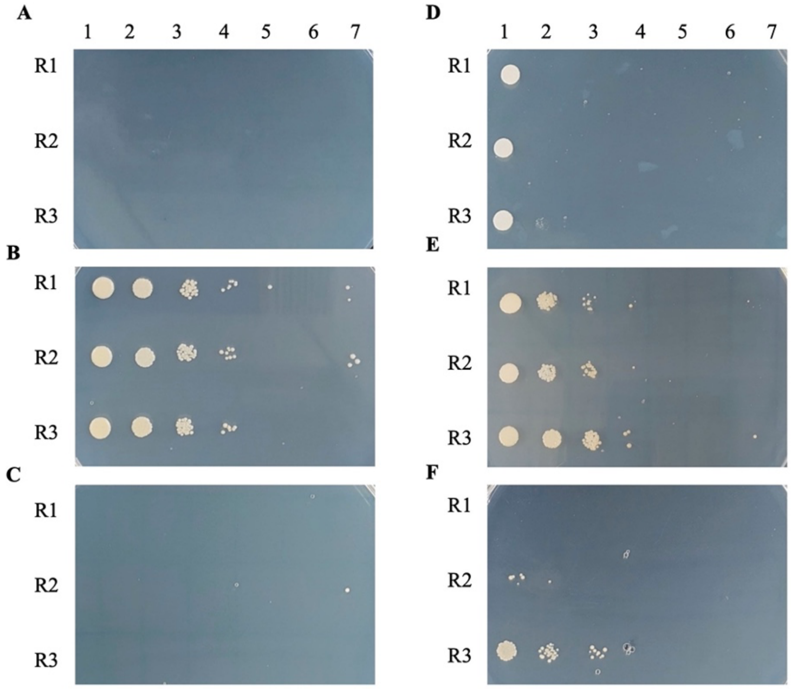

Supplementary Figure 1. The growth of yeast *K. phaffii* CBS 7435 on minimal media supplemented with sodium cyanate (NaOCN). The growth of the yeast on different plates containing M2 media, 1.5% agar, 5 $g.L^{-1}$ methanol, and the following nitrogen sources: (A) M2 agar without $(NH_4)_2HPO_4$, (B) complete M2 agar including 3.15 $g.L^{-1}$ $(NH_4)_2HPO_4$, (C) M2 agar with 1mM NaOCN (without $(NH_4)_2HPO_4$), (D) M2 agar with 2.5 mM NaOCN (without $(NH_4)_2HPO_4$), (E) M2 agar with 5 mM NaOCN (without $(NH_4)_2HPO_4$), (F) M2 agar with 10 mM NaOCN (without $(NH_4)_2HPO_4$). Overnight-grown yeast cultivation in YPD media was collected and washed with distilled water, then was adjusted to the OD of 0.6. This was used as the initial inoculum, which was further serially diluted, finally 10 µL of each sample was spotted on plates. 1-7 shows the $1,10^{-1},10^{-2},10^{-3},10^{-4},10^{-5},10^{-6}$ dilutions respectively. R1, R2, and R3 show three biological replicates. All the experiments have been done in technical triplicates.

1.2 Supplementary Tables

Supplementary Table 1. Ultimate and proximate analyses of typical softwood pellets

**Parameter**	**Softwood pellets**
Water content [wt.-%]	7.2
Ash content [wt.-%$_{db}$]	0.2
Carbon [wt.-%$_{daf}$]	50.8
Hydrogen [wt.-%$_{daf}$]	5.9
Nitrogen [wt.-%$_{daf}$]	0.2
Sulfur [wt.-%$_{daf}$]	0.005
Chlorine [wt.-%$_{daf}$]	0.005
Oxygen[a] [wt.-%$_{daf}$]	43.1
Volatile matter [wt.-%$_{daf}$]	85.6
LHV [MJ/kg$_{db}$]	18.9
Ash deformation temp. [°C]	1330
Ash flow temp. [°C]	1440

[a] Calculated by difference to 100 wt.-%$_{daf}$

Supplementary Table 2. Operating parameters of the DFB gasifier and the biodiesel scrubber; **LGR**, lower gasification reactor, **UGR** upper gasification reactor, **CR** combustion reactor

**Parameter**	**Value**
**DFB gasifier**	
Bed material mixture [wt.-%]	80/20 olivine/limestone mixture
Feedstock	Softwood pellets
Mean temperature LGR [°C]	836
Temperature UGR [°C]	962
Mean temperature CR [°C]	1015
Fuel input LGR [kW]	91
Fuel input CR [kW]	52
Steam-to-fuel ratio [$kg_{H2O}/kg_{fuel,daf}$]	0.80
**Biodiesel scrubber**	
Particle filter temperature [°C]	258
Scrubber gas entry temperature [°C]	214
Scrubber gas exit temperature [°C]	18
Scrubber solvent to gas ratio [kg_{RME}/kg_{PG}]	127
